# Biological functions of natural antisense transcripts

**DOI:** 10.1186/1741-7007-11-31

**Published:** 2013-04-12

**Authors:** Andreas Werner

**Affiliations:** 1 RNA Interest Group, Institute for Cell and Molecular Biosciences, Newcastle University, Framlington Place, Newcastle upon Tyne, NE2 4HH, UK

## Abstract

In theory, the human genome is large enough to keep its roughly 20,000 genes well separated. In practice, genes are clustered; even more puzzling, in many cases both DNA strands of a protein coding gene are transcribed. The resulting natural antisense transcripts can be a blessing and curse, as many appreciate, or simply transcriptional trash, as others believe. Widespread evolutionary conservation, as recently demonstrated, is a good indicator for potential biological functions of natural antisense transcripts.

See research article: http://www.biomedcentral.com/1471-2164/14/243

## 

By the mid-1980s antisense transcription in mammalian genomes had already been described by a few isolated reports. Despite the recognized regulatory potential of complementary RNA, antisense transcription in mammals was long perceived as a biological oddity. This perception started to change at the beginning of the genomic era when pioneering data mining efforts and the sequencing of large cDNA datasets revealed significant numbers of antisense transcripts. It is now accepted that a significant proportion of genomic loci in mammalian genomes - for example, 40% in human and 72% in mouse - are transcribed in both directions [1]. The discrepancy between human and mouse is more a reflection of the fact that antisense transcription has been studied in much greater detail in mouse than in human rather than a 'real' biological difference.

Sense and antisense transcript pairs come in various forms depending on the exact location of the complementary overlap and the processing of the transcripts. The most common meaning of the term 'antisense transcript' refers to a protein coding sense transcript and a fully processed (capped, polyadenylated) antisense RNA with complementarity in exonic regions (Figure 1). The key issue of whether the antisense transcripts act as exquisitely specific gene regulators or are simply transcriptional waste that a cell has learned to live with is still a matter of controversy [2]. However, a study published in *BMC Genomics* reports conservation of natural antisense transcripts at a large scale between human, rat and mouse, which strongly suggests that there is biological sense to having antisense transcripts [3].

**Figure 1:**
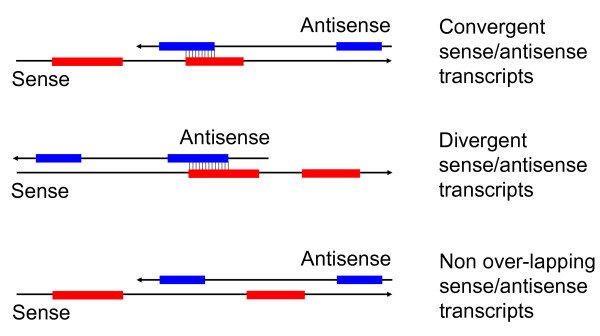
Schematic arrangements of transcripts from bi-directionally transcribed genes (sense exons are in red, antisense exons are in blue).

## Regulatory antisense transcripts

The most convincing way to demonstrate the biological significance of an antisense transcript is to interfere with its expression and demonstrate phenotypic consequences or altered expression levels of the sense transcript. This approach has been pursued to investigate a still limited but increasing number of antisense transcripts. The best characterized examples include *Airn*, *Kcnq1ot1* and *Tsix*, antisense transcripts involved in parental imprinting (*Airn*, *Kcnq1ot1*) and X chromosome inactivation (*Tsix*). Expression of *Airn*, *Kcnq1ot1* and *Tsix* induces allele-specific chromatin changes and eventually leads to the silencing of the cognate sense transcript.

Parental imprinting and X chromosome inactivation are epigenetic phenomena prominently observed in mammals but absent or mechanistically distinct in other animals such as birds or insects [4]. In human and mouse, however, there are well documented examples of antisense-induced chromatin modifications, suggesting that antisense RNA-guided gene silencing could well have broader significance. For example, a rare form of inherited α-thalassemia is caused by ectopic expression of an antisense transcript. It originates from the constitutively active *LUC7* gene, which is brought into close vicinity of the *HBA2* gene by a gene deletion. The *LUC7* transcript (antisense to *HBA2*) triggers the methylation of the *HBA2* promoter, the silencing of the protein coding sense gene and, therefore, a reduction of α-hemoglobin [5]. Another antisense transcript has been linked in mouse to heterochromatin formation and concomitant silencing of the tumor suppressor gene p15, associated with a variety of cancers [6].

The fact that sense and antisense transcripts share complementary regions, as well as extensive indirect evidence that sense and antisense transcripts may be co-expressed in the same cell, suggests the formation of sense-antisense RNA hybrids. Such an assumption comes, however, with a considerable reservation: double-stranded RNA molecules in the cytoplasm are a signal of viral infection that activates protein kinase R and the interferon pathway, eventually triggering an immune response. How this might be avoided is unclear, but despite this concern, a growing number of antisense transcripts have been shown to regulate the expression of their cognate sense transcripts through mechanisms based on RNA-RNA interaction. Interesting examples include the bi-directionally transcribed genes encoding HIF-1α (hypoxia-induced factor 1α) and β-secretase, where the antisense transcripts have a stabilizing effect on the protein coding sense transcript. This is achieved by either blocking an RNA destabilizing motif in *HIF*-*1α* mRNA or competing for a microRNA site in β-secretase mRNA [7,8]. Such RNA-masking could well be of general significance since many sense-antisense pairs overlap at their 3’ ends, where both stability motifs and microRNA binding sites are predominantly situated.

**Figure 2:**
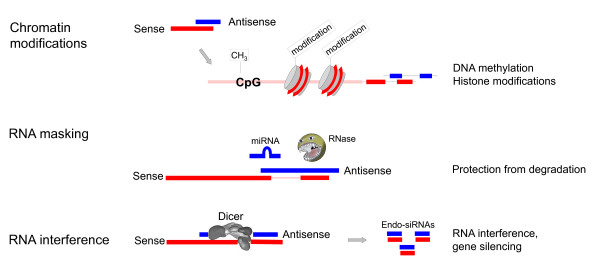
**Established cellular mechanisms related to the transcription of natural antisense transcripts. **Top panel: over-expression of an antisense transcript (in blue) causes the modification and concomitant silencing of the sense promoter. The exact mechanism of how the repressive chromatin marks are established is yet unknown. Middle panel: in RNA masking, the antisense transcript directly interacts with the sense transcript (in red) and occludes regulatory sequences. Bottom panel: in RNA interference, sense and antisense transcripts hybridize and the double-stranded RNA is further processed by the RNA interference-linked enzymatic machinery. This may lead to post-transcriptional or transcriptional gene silencing. endo-siRNA, endogenous small interfering RNA; miRNA, microRNA.

An unresolved problem regarding stoichiometric interactions between the complementary transcripts is represented by the observation that antisense RNAs are generally expressed at much lower levels than protein coding transcripts. Alternatively, sense/antisense hybrids could be processed by the RNA interference pathway and lead to transcriptional or post-transcriptional effects. As attractive as such a scenario would appear, there is little evidence for endogenous small interfering RNAs derived from complementary sense/antisense transcripts (genic endo-siRNAs). Large scale sequencing experiments have revealed some genic endo-siRNAs and also a specific gene, *Slc34a1* (encoding a sodium/phosphate transport system), has been shown to produce them in selected tissues [9]. However, a link between RNA interference and antisense transcription seems to represent an exception in somatic cells and only to occur in restricted cell populations (Figure 2).

To summarize, a growing number of antisense transcripts with an established function have been identified but still many questions remain concerning their mechanisms of action. Progress is rather slow because of formidable experimental difficulties inherent to antisense research. To start, most of the antisense transcripts are expressed at very low levels and are difficult to detect on northern blots or by *in situ* hybridization. Amplification then carries the risk of losing the orientation and swapping sense for antisense. More significantly, the intricate relation between sense and antisense transcription means that experimental perturbation of one transcript inevitably interferes with the expression of the other. Whether documented changes in expression then have biological significance is much harder to demonstrate. The comparative study represented by the *BMC Genomics* paper by Ling *et al*. [3] thus provides a valuable resource to assess the potential importance of newly identified antisense transcripts. Phylogenetic conservation is a strong indicator of physiological relevance and the presented data will help to make informed decisions about whether a specific antisense transcript warrants further investigation.

## A grand design?

Research concentrating on single loci and regulatory interactions between sense and antisense transcripts resembles the proverbial focus on trees without considering the forest. Interestingly, there is indeed compelling evidence that something like a grand design behind antisense transcription exists, which concurs well, for example, with the conserved expression of natural antisense transcripts in mammals. This evidence comes from the observation that antisense transcripts are significantly under-represented on mouse and human X chromosomes compared to autosomes (this bias is not found in flies, which keep both X chromosomes active in XX females and upregulate X-specific gene expression in XY males). Moreover, it has been reported that the expression of natural antisense transcripts correlates with random imprinting or random monoallelic gene expression [9]. All these observations point to a scenario where antisense transcription may induce allele-specific silencing of protein coding sense transcripts, likely on a considerable scale but only in specialized tissues. The potential of antisense transcripts to induce gene silencing in this way would be detrimental on X chromosomes because usually only one copy of an X-linked gene is active and antisense-induced silencing would result in its complete knockout: this could explain why natural antisense transcripts are significantly under-represented on the X chromosome. Other hypotheses have also been proposed to explain the biological benefit of antisense transcription: for example, because antisense transcripts have short introns they have been hypothesized to favor rapid RNA synthesis and a short induction time [10].

The general expression patterns reported by Ling *et al*. suggest that the brain and testis are hotspots of antisense transcription in mouse and human. Interestingly, in both organs transposons play important roles: in brain, transposons are thought to add a creative touch to learning, and in testis they help an organism to quickly adapt to a changing environment. Retrotransposition, however, also comes with the danger of randomly damaging vital genes. An admittedly speculative but highly intriguing hypothesis suggests that natural antisense transcripts may quality control the transcriptional output after an episode of retrotransposition. Accordingly, antisense-induced silencing would target damaged genes and purge the transcriptome of deleterious transcripts [11].

To conclude, the current understanding of natural antisense transcripts is incomplete but antisense RNA starts to make sense both as a regulator of individual genes and also as a genomic organizer on a larger scale.
